# Pharmacological Management and Longitudinal Cognitive, Psychiatric, and Behavioral Changes During Stem Cell Therapy in a Patient With Mesial Temporal Sclerosis: A Case Report

**DOI:** 10.7759/cureus.111353

**Published:** 2026-06-23

**Authors:** Eduardo Ciarrochi, Juan Bautista Ciarrochi, Leonardo Adrian Ugartemendía

**Affiliations:** 1 Regenerative Medicine, Clínica de Cirugía Especializada, Buenos Aires, ARG

**Keywords:** epilepsy, mesial temporal sclerosis, multidisciplinary management, polypharmacy, stem cell therapy

## Abstract

Polypharmacy is common in epilepsy, particularly in patients with psychiatric comorbidities requiring concomitant pharmacological treatment. Long-term treatment often increases the risk of adverse drug reactions and pharmacological interactions.

Stem cell therapy has emerged as a topic of growing interest in neurology because of its immunomodulatory and neuromodulatory properties.

We report the case of a 15-year-old female patient with mesial temporal sclerosis characterized by marked cognitive and psychiatric comorbidities requiring a complex five-drug pharmacological regimen. The patient underwent treatment with autologous stem cells harvested from the superficial abdominal fascia and was followed clinically for one year.

During follow-up, the patient also continued to receive antiepileptic treatment, psychopharmacological management, psychotherapy, and multidisciplinary clinical care. Throughout follow-up, individualized pharmacological optimization was performed under close clinical monitoring.

This case highlights the importance of individualized pharmacological management during longitudinal multidisciplinary care. The report describes the longitudinal clinical course and pharmacological optimization of a patient with mesial temporal sclerosis undergoing stem cell therapy within a broader multidisciplinary treatment framework.

## Introduction

Polypharmacy is defined as the concurrent use of multiple medications to treat a medical condition. The combination of drugs both from the same class and from different pharmacological classes may lead to various pharmacodynamic and pharmacokinetic interactions [1]. The occurrence of adverse drug reactions is one of the main risks in patients treated with multiple drugs. The incidence of these reactions is close to 40% in patients treated with five drugs and exceeds 80% in patients taking seven or more drugs [2].

The prevalence of psychiatric disorders in patients with epilepsy is high, with up to 62% prevalence of mood disorders and up to 9% prevalence of psychosis [3].

Mesial temporal sclerosis (MTS) is a well-defined entity of structural focal epilepsy (epilepsy resulting from an identifiable structural brain abnormality) characterized by gliosis and neuronal loss in the hippocampus and adjacent structures [4]. Seizures associated with MTS are distinguished by their complexity and clinical heterogeneity, involving sensory, mnestic, and motor symptoms [5].

Immune dysregulation has been increasingly recognized in patients with epilepsy, with elevated levels of cytokines such as IL-1B, IL-18, CCL2, CCL3, and CCL4, suggesting that inflammation is an important component of epileptogenesis [6]. Furthermore, excitotoxicity and glutamate metabolism have been shown to play significant roles in the disease-induced damage. A key enzyme involved in maintaining this balance is glutamine synthetase, which is expressed in astrocytes. Reduced expression of this enzyme has been observed in epileptic patients as a consequence of deregulation of the Hippo-yes-associated protein (YAP) signaling pathway, leading to the extracellular accumulation of glutamate, which ultimately contributes to neuronal death [7]. Likewise, impaired astrocytic function affects neuronal development, synaptogenesis, and neurogenesis [8].

The Hippo-YAP signaling pathway plays a critical role in controlling organ size and tissue regeneration. Multiple studies demonstrate that the Hippo signaling pathway is involved in neuroinflammation, neuronal cell differentiation, and neuronal death [9].

The application of stem cells in neurological conditions represents a rapidly expanding field. Stem cells are an attractive option for use in the treatment of epilepsy because of their potential to replace damaged cells, restore cognitive function, and stabilize focal electrical activity [10]. Stem cells are undifferentiated cells with the capacity for self-renewal and differentiation into various types of tissues. They interact closely with the immune system and can act on the inflammatory microenvironment present in various medical conditions [11].

The objective of this study is to describe the pharmacological management and optimization of treatment in a patient with MTS undergoing longitudinal multidisciplinary management that included autologous stem cell therapy. In addition, we describe the main clinical findings observed during a one-year follow-up period and the gradual adjustments made to the patient's pharmacological regimen.

## Case presentation

We present the case of a 15-year-old female patient diagnosed with MTS. The patient had no significant family medical history.

According to the mother, early neurodevelopmental milestones were achieved within normal limits: the patient sat independently at seven months, crawled at eight months, and achieved independent walking at 16 months, followed by appropriate bladder and bowel control at two years of age. Throughout her childhood, she experienced febrile seizures and was diagnosed with epilepsy in 2022.

Regarding her academic history, the patient entered kindergarten at age 3. According to the mother, the initial school environment had a high student-to-teacher ratio, which hindered the patient's learning progress. Consequently, during the seventh grade, the family decided to transfer her to a different educational institution that offered more personalized teaching. The patient presented pre-critical, critical, and post-critical manifestations. Episodes of unexplained fear, headaches, autonomic phenomena, and behavioral arrests characterized the patient's pre-critical manifestations. Among these manifestations, the behavioral arrests and the emotional component were the most intense. Her seizures were primarily characterized by both psychiatric and motor components; specifically, manifestations such as auditory hallucinations (hearing unpleasant voices), motor automatisms, and absence seizures defined her clinical presentation. Among these manifestations, the auditory hallucinations and absence seizures were the most intense. Post-critical manifestations were characterized by post-ictal fatigue and confusion.

At stem cell treatment initiation in May 2025, the patient's family helped her with her medication regimen, which consisted of five drugs (Table 1). At that moment, the patient had been on that medication regimen since October 2024.

**Table 1:** Pharmacological regimen at treatment initiation

Drug	Dose
Lamotrigine, 50 mg tablets	50 mg in the morning/50 mg in the evening
Clonazepam, 2.5 mg/mL oral drops	Three drops in the morning, two drops in the afternoon, five drops in the evening
Risperidone, 0.1% oral drops	Five drops in the morning, five drops in the evening
Quetiapine, 25 mg tablets	25 mg in the morning/25 mg in the evening
Biperiden, 2 mg tablets	4 mg once daily

Regarding the patient's recent clinical history, the family reported a hospitalization due to psychotic depression seven months before treatment initiation. She required inpatient psychiatry hospitalization due to a severe depressive episode with psychotic symptoms (ICD-10: F32.3). The psychiatric admission lasted one month, following which she achieved clinical stabilization and was medically discharged.

One year before treatment initiation, a psychometric assessment was conducted in a specialized institution. The battery administered included the following standardized instruments classified by cognitive domain: for intellectual function, Wechsler Preschool and Primary Scale of Intelligence (WPPSI); for comprehensive neuropsychological function, Child Neuropsychological Assessment (Evaluación Neuropsicológica Infantil (ENI)) and the Developmental Neuropsychological Assessment, Second Edition (NEPSY-II); for attention and executive function, d2 Test of Attention, Trail Making Test (TMT), and the Behavior Rating Inventory of Executive Function, Second Edition (BRIEF-2); for visuospatial and memory function, Rey-Osterrieth Complex Figure (ROCF) and the Test of Verbal Learning and Memory (Test de Aprendizaje y Memoria Verbal Infantil (TAMV-I)); for language and speech, Comprehensive Speech and Language Evaluation; for behavioral and emotional profile, System for the Evaluation of Children and Adolescents (Sistema de Evaluación de Niños y Adolescentes (SENA)); and for adaptive behavior, Adaptive Behavior Assessment System, Second Edition (ABAS-II).

Regarding her pre-treatment cognitive profile, the patient exhibited an overall very low performance, with a Full Scale IQ (FSIQ) of 68, which places her within the range of an intellectual disability. Her greatest strength was noted in the Fluid Reasoning Index (FRI: 94), which fell within the average range. Conversely, the Visual-Spatial Index (VSI: 72) and the Working Memory Index (WMI: 72) were in the low range. Finally, the Processing Speed Index (PSI: 66) and the Verbal Comprehension Index (VCI: 59) scored significantly below the mean, underscoring a severely depressed cognitive baseline. Qualitatively, a pronounced slowing of task execution was observed, along with substantial difficulties in comprehending and responding to questions. Despite these specific language-based impairments, no statistically significant discrepancy was found between her verbal and non-verbal performances, indicating a global cognitive impairment.

During this first encounter with the patient, she was admitted with minimal communicative intent, establishing contact only with her sister, who responded on her behalf. During the initial assessment, the physical examination and anamnesis were highly challenging, and the Mini-Mental State Examination (MMSE) could not be performed due to the patient's refusal and severely scattered attention. Furthermore, she presented as overtly guarded and mistrustful toward the examiners, demonstrating a complete lack of communicative intent.

At the visit, we dealt with a patient with significantly impaired cognitive functions such as memory, attention, language, gnosis, and thinking; these cognitive impairments affected her ability to perform daily activities as well as her academic performance.

Regarding her academic and behavioral performance, the patient exhibited a severely short attention span, which significantly hindered her academic progress in class. She experienced significant difficulties completing homework assignments, thereby requiring continuous adult supervision. Socially, the family indicated that the patient was naturally inclined to socialize and establish peer relationships, often preferring to interact with younger children and maintaining a small circle of close friends. Despite her willingness to socialize, her family noted that she frequently experienced social isolation by her school peers. The patient's reading accuracy was borderline to average, primarily due to errors during reading aloud. However, more pronounced difficulties were identified in reading comprehension, with scores falling significantly below the mean. This underscores substantial challenges in extracting and retaining critical information from written text, as well as in following conditional instructions. In the language evaluation, the patient exhibited significant difficulties in identifying age-appropriate vocabulary relative to her chronological age, as well as challenges in lexical categorization, sentence recall, and the comprehension of textual details and concepts. Regarding her expressive language level, she demonstrated pronounced difficulties in communicating word associations, solutions, and deductions, reflecting a significant developmental delay for her age.

Furthermore, she demonstrated behavioral challenges, including non-compliance with school regulations and interpersonal conflicts with both teachers and classmates. During clinical evaluation, the patient expressed significant school refusal, stating a strong aversion to attending school.

The most significant cognitive limitations were in attention and memory. Regarding attention, it was very difficult to maintain a coherent conversation with the patient, as she was easily distracted and often ignored what the examiner was telling her. This impairment greatly limited her school performance. The patient was unable to retain and recall recent events, such as words or numerical sequences the examiner asked her to repeat. During visits before treatment, the patient frequently confused examiners with other individuals and repeatedly introduced herself during the visits as if she had never met the medical team before. Mnestic and psychiatric symptoms deeply affected her normal psychosocial and educational development.

According to the official radiological report, the magnetic resonance scan obtained before treatment initiation demonstrated the following: "bilateral dilation of the temporal horns, accompanied by a decrease in the volume of the hippocampi, predominantly on the right side". A pre-treatment coronal T2-weighted fluid-attenuated inversion recovery magnetic resonance imaging (T2-FLAIR MRI) sequence of the hippocampal region, with imaging findings consistent with right-sided MTS, is shown in Figure 1.

**Figure 1:**
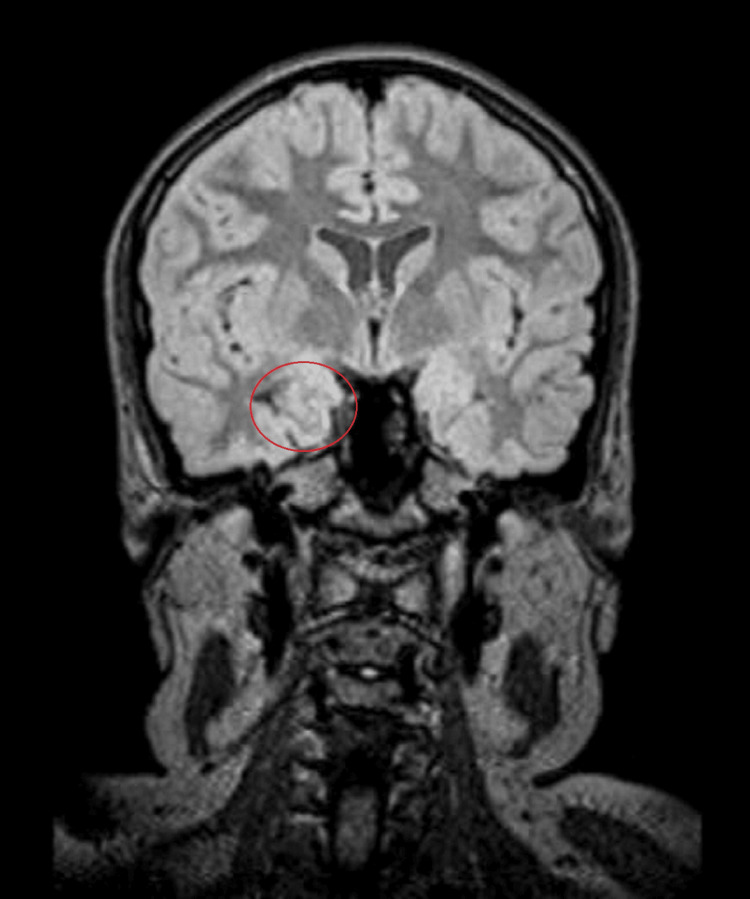
Pre-treatment coronal T2-FLAIR MRI sequence showing the approximate hippocampal region (circle), with imaging findings consistent with right-predominant mesial temporal sclerosis and definite temporal horn asymmetry T2-FLAIR MRI: T2-weighted fluid-attenuated inversion recovery magnetic resonance imaging

The patient's family expressed concern about her medication regimen and expected drug doses to be reduced or all drugs discontinued after the initiation of stem cell therapy. It was explained that adjustments to the medication regimen could be made based on the patient's clinical progress; however, complete discontinuation of all drugs was not considered appropriate.

The patient and her parents were self-referred and traveled from their home country, Ecuador, specifically to our institution in Argentina. Their decision was prompted by a word-of-mouth recommendation from another patient previously treated by our team. The family's primary therapeutic objective focused on addressing the patient's severe cognitive and neuropsychiatric symptomatology, combined with a strong preference to avoid invasive neurosurgical interventions. After discussion with the patient's family and considering the available preclinical and clinical literature regarding stem cell therapy, it was decided to proceed with autologous stem cell treatment.

Prior to the initiation of the autologous stem cell therapy, a complete blood count and a coagulation profile were performed. Both evaluations were unremarkable and within normal limits. Autologous stem cells were harvested from the superficial abdominal fascia and cultured for subsequent use. The procedure and application of the stem cell concentrate were performed successfully, and no adverse events were reported. The entire intervention was conducted on an outpatient basis. Following a two-day rest period, the stem cell concentrate was successfully applied over four consecutive days, and no adverse events were reported in this first stage of treatment. Following the initial treatment phase, the patient returned to her country. Clinical follow-up assessments and subsequent stem cell applications were scheduled at monthly intervals in agreement with the patient's family. This second therapeutic phase was maintained until May 2026.

Throughout the follow-up period, the patient remained under multimodal management that included antiepileptic medication, psychopharmacological treatment, psychotherapy, regular physical activity, multidisciplinary clinical follow-up, and stem cell therapy.

During the follow-up period, changes in epilepsy-related symptoms were documented through clinical assessments and family reports. Pre-critical manifestations were among the earliest symptoms in which changes were noted. According to family reports and clinical follow-up assessments, headaches and autonomic symptoms were experienced less frequently during follow-up. In addition, the patient no longer reported the distressing auditory hallucinations that had previously caused significant anxiety and emotional distress.

At the beginning of follow-up, absence seizures were among the most frequently reported manifestations. These episodes were characterized by brief periods during which the patient would stare fixedly at a point for several seconds and subsequently remain unaware of the event. According to longitudinal clinical documentation and family reports, the frequency of these episodes progressively decreased during the follow-up period.

According to reports from both the family and the clinical team, the patient has remained seizure-free since September 2025. During this period, the patient was described as carrying out her academic and daily activities with greater independence, confidence, and stability. These observations were consistently documented throughout follow-up by both the patient's family and the treating medical team.

Changes in cognitive domains including memory, attention, and language were documented during subsequent clinical follow-up. The patient described her daily activities with greater fluency and coherence, using a wider range of words and expressions. She also discussed past experiences and events from previous visits and recalled distant memories during clinical encounters.

During follow-up, the patient was observed expressing personal interests, engaging in age-appropriate and consistent conversations, and using a broader vocabulary with more detailed descriptions. She shared her future aspirations and discussed the profession she would like to pursue as an adult. In addition, she became increasingly interested in the people around her, frequently asking members of the medical team about their work and daily activities.

The patient participated in regular physical training sessions with a personal trainer, which she described as highly motivating and enjoyable. The patient and her family reported that she became less socially withdrawn and suspicious and interacted more frequently with peers of her own age.

According to family reports, episodes of classroom conflict and interpersonal disputes became less frequent. The patient also began to show interest in certain school subjects, a behavior not previously observed. The reduction in seizure frequency, together with the cognitive and behavioral changes documented during follow-up, was associated with greater participation in daily activities, improved classroom engagement, increased involvement in exercise routines, more frequent peer interactions, and the ability to sustain activities for longer periods.

The patient reported positive changes in social, educational, and daily-life functioning during follow-up. Similar observations were also reported by her family.

A follow-up MRI obtained in June 2025 is shown in Figure 2 and Figure 3. According to the official radiological report, the magnetic resonance study was interpreted as normal.

**Figure 2:**
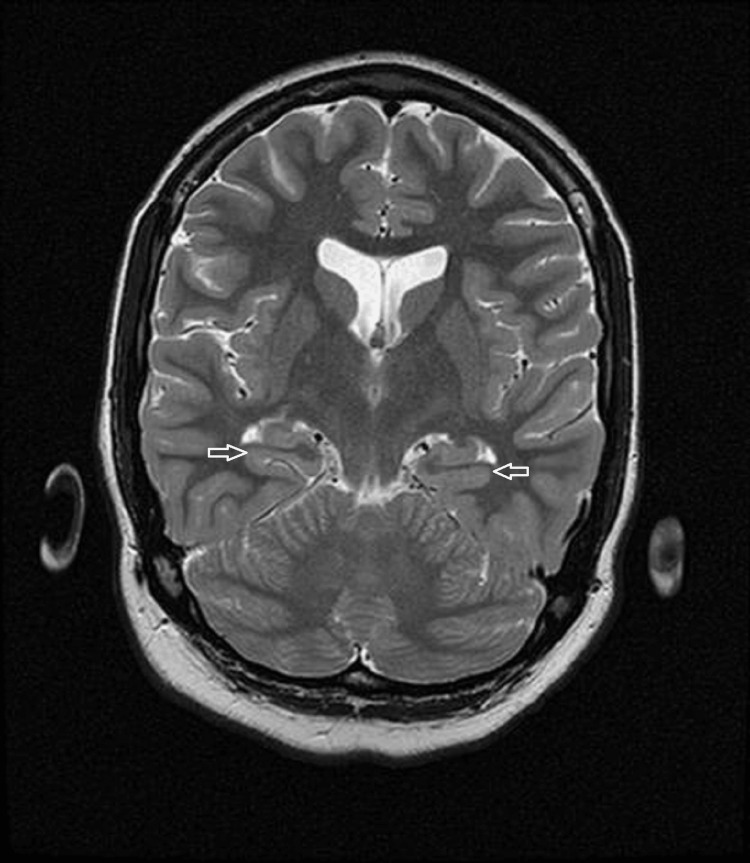
Follow-up coronal T2-weighted FSE sequence showing the hippocampal regions (arrows), with preserved hippocampal morphology and no obvious signal abnormalities FSE: fast spin echo

**Figure 3:**
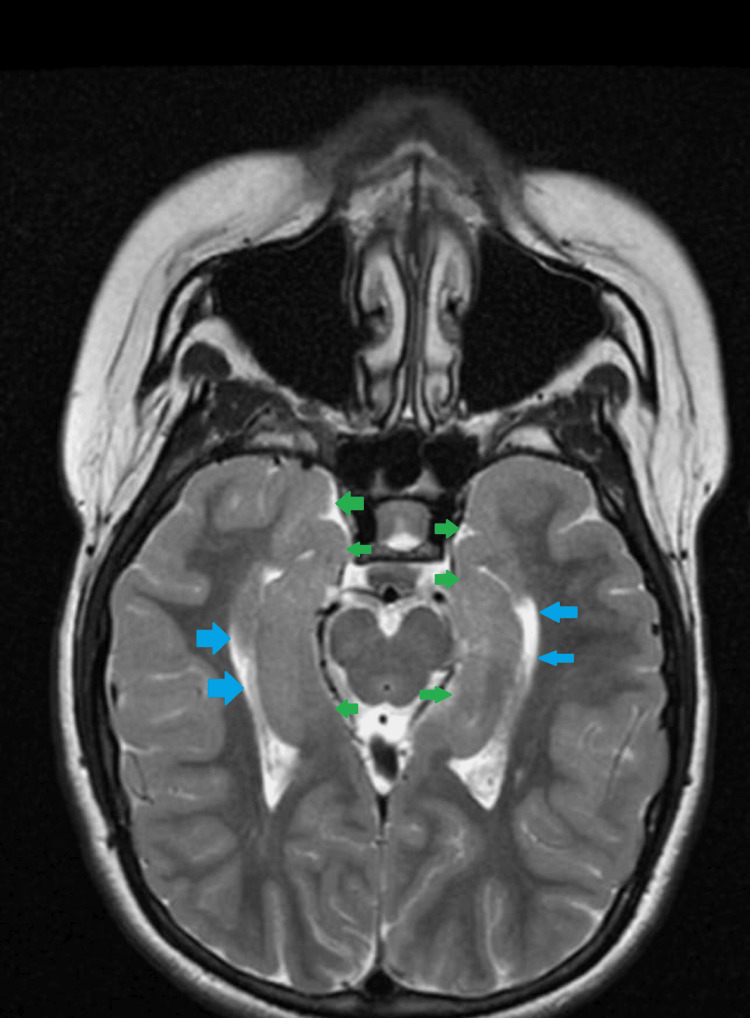
Follow-up axial T2-weighted MRI sequence demonstrating the symmetric appearance of the mesial temporal regions (green arrows) and non-dilated temporal horns (blue arrows) MRI: magnetic resonance imaging

Following a period of sustained clinical stability, and in consultation with the patient and her family, it was decided to adjust the patient's medication regimen. It was explained that these adjustments would be made one medication at a time, accompanied by strict de-escalation schedules. Specific withdrawal warning signs for each medicine were also provided. It was again emphasized that the objective was not the complete withdrawal of all medications, but rather the rational and personalized optimization of the pharmacological regimen.

The first medication to be adjusted was biperiden 2 mg, which had been prescribed to prevent extrapyramidal symptoms associated with oral risperidone therapy. The patient was receiving 4 mg/day (two 2 mg tablets administered in the morning). A four-week tapering schedule was implemented as follows: 3 mg/day during week 1, 2 mg/day during week 2, 1 mg/day during week 3, and complete discontinuation during week 4. The tapering process was well tolerated, and no adverse events were observed.

Following this, tapering of the afternoon clonazepam dose (two drops of a 2.5 mg/mL solution) was initiated. The patient's family was provided with specific warning signs to monitor during withdrawal, including anxiety, headaches, seizures, insomnia, and other potential withdrawal-related symptoms. The dose was reduced to one drop daily during weeks 1 and 2 and then discontinued during weeks 3 and 4, with the patient closely monitored by her family for potential adverse events. The tapering process was well tolerated, and no adverse events were observed.

Finally, a stepwise withdrawal of quetiapine was performed. The patient was initially receiving 25 mg in the morning and 25 mg in the evening. A structured four-week tapering schedule was implemented as follows: 12.5 mg in the morning and 25 mg in the evening during week 1, 25 mg in the evening only during week 2, 12.5 mg in the evening during week 3, and complete discontinuation during week 4. The tapering schedule was well tolerated, and no adverse events were observed.

The patient is currently on a three-drug regimen: lamotrigine, risperidone, and clonazepam (Table 2).

**Table 2:** Current pharmacological regimen after one year of clinical follow-up

Drug	Dose
Lamotrigine, 50 mg tablets	50 mg in the morning/50 mg in the evening
Clonazepam, 2.5 mg/mL oral drops	Three drops in the morning, five drops in the evening
Risperidone, 0.1% oral drops	Five drops in the morning, five drops in the evening

At the last visit, the patient reported that before treatment, she was feeling absent-minded and disconnected from both family and friends, being unaware of her condition.

The patient reported forming new friendships since the beginning of the current school year and described feeling happier with her social environment. She discussed her school subjects in detail, particularly biological sciences, and appeared increasingly engaged in academic activities. Notably, she expressed concern about missing classes for medical appointments and requested that future visits be scheduled outside school days whenever possible, a behavior not observed prior to treatment.

The patient also reported a more active daily routine, including regular physical exercise and participation in a wider range of activities. She reported being able to celebrate her birthday without fear of experiencing seizures, which represented a significant change from previous years. Despite these changes, some challenges remain, including adherence to healthy dietary habits and a more comprehensive understanding of complex social situations and past life events.

In addition to regular follow-up with our team, the patient currently attends supervised exercise sessions twice weekly and participates in weekly cognitive-behavioral therapy.

At present, both the patient and her family continue to report positive changes in social interaction, school participation, and overall quality of life.

## Discussion

Beyond seizure control, several biological mechanisms have been implicated in the pathophysiology of epilepsy, including neuroinflammation, astrocytic dysfunction, alterations in glutamate metabolism, and cellular signaling pathways such as the Hippo-YAP pathway [6-9]. These processes have been proposed as contributors to neuronal dysfunction and structural changes observed in epilepsy. In this context, stem cells and their exosomes have attracted growing research interest because of their reported immunomodulatory and neuromodulatory properties [10,11].

Proposed goals of stem cell-based therapy in epilepsy include replacing lost cells, reconstructing impaired brain circuits, and using these cells as carriers of endogenous neurotrophic compounds. In our case report, we used autologous stem cells. These cells do not pose a particular ethical problem and do not interfere with the recipient's immune system [12].

Experimental studies have suggested that glutamatergic dysfunction, neuroinflammation, and astrocytic alterations may contribute to epileptogenesis. Stem cells and their exosomes have attracted research interest because of their proposed ability to modulate some of these processes in preclinical models [8,10,13-15]. However, patients with epilepsy are often managed with complex polypharmacy regimens that require careful longitudinal review. In this context, the present case highlights the importance of longitudinal multidisciplinary management, in which pharmacological treatment, stem cell therapy, psychotherapy, and physical activity were implemented concurrently and adjusted according to the patient's clinical course.

Throughout follow-up, gradual pharmacological adjustments were performed based on the patient's clinical course. Biperiden 4 mg/day, quetiapine 25 mg morning/25 mg evening, and a low-dose afternoon clonazepam regimen were progressively tapered, and no adverse events were observed.

Biperiden had originally been prescribed to prevent extrapyramidal symptoms associated with risperidone treatment. However, the prophylactic use of biperiden is generally not recommended, long-term administration beyond several weeks is discouraged, and pediatric experience remains limited [16,17]. The use of two drugs of the same class, such as quetiapine and risperidone (both second-generation antipsychotics), increases the risk of drug adverse reactions and toxicity [1]. Given the patient's sustained clinical stability during follow-up and the objective to reduce polypharmacy, it was decided to gradually discontinue quetiapine while maintaining risperidone therapy.

At the same time, treatment with lamotrigine, risperidone, and clonazepam was continued according to the patient's clinical status and longitudinal follow-up findings. Medication adjustments were individualized with the goal of maintaining clinical stability while reducing unnecessary polypharmacy whenever possible.

Limitations and future directions

The primary limitation of this report is that it describes the clinical course of a single patient with a one-year follow-up period. Furthermore, multiple interventions were implemented concurrently throughout this timeframe, including antiepileptic treatment, psychopharmacological management, psychotherapy, supervised physical activity, multidisciplinary clinical follow-up, and stem cell therapy. Therefore, the clinical changes documented in this case cannot be attributed exclusively to stem cell therapy, and the relative contribution of each therapeutic intervention remains uncertain. Although the clinical evolution observed in this patient is encouraging, these findings should be interpreted with caution.

Another limitation is that standardized neuropsychological assessments were not repeated during follow-up. Consequently, longitudinal changes in cognitive and behavioral domains were evaluated primarily through clinical observations and reports provided by the patient, her family, and the treating medical team. Future studies incorporating repeated standardized assessments may allow a more objective evaluation of longitudinal clinical changes.

Despite these limitations, the present report possesses noteworthy strengths. The case provides a longitudinal description of a patient with MTS managed through a multidisciplinary treatment strategy that included individualized pharmacological optimization, psychotherapy, physical activity, and stem cell therapy over an extended follow-up period. Importantly, no treatment-related adverse events were observed throughout follow-up. Moreover, clinical changes were documented across cognitive, educational, social, and daily-life domains through regular clinical evaluations involving the patient, her family, and the treating physicians. This case highlights the potential coexistence of regenerative medicine approaches and rational pharmacological management, emphasizing that stem cell therapy should not necessarily be viewed as a replacement for conventional treatment. Rather, within the context of this case, stem cell therapy is best interpreted as one component of a broader multimodal treatment strategy that included pharmacological treatment, psychotherapy, physical activity, and multidisciplinary clinical follow-up.

Future research should focus not only on evaluating the safety and efficacy of stem cell-based therapies in epilepsy but also on establishing standardized treatment protocols and outcome measures. Particular attention should be directed toward defining patient selection criteria, indications and contraindications, age-related considerations, cell dosing strategies, treatment duration, and long-term follow-up parameters. Prospective controlled studies with larger patient cohorts and longer follow-up periods will be essential to better characterize the role of regenerative medicine within multimodal treatment strategies for epilepsy and to clarify the relative contribution of individual therapeutic interventions to patient outcomes.

## Conclusions

This case highlights the importance of individualized pharmacological management in a patient with MTS undergoing stem cell therapy within a broader multimodal treatment strategy. Throughout the follow-up period, clinical changes were documented across neurological, cognitive, behavioral, social, and educational domains, while gradual pharmacological optimization was achieved without treatment-related adverse events.

Given the observational nature of a single case report and the concurrent implementation of multiple therapeutic interventions, these findings should be interpreted with caution. Nonetheless, this report illustrates the feasibility of integrating regenerative medicine approaches with rational pharmacological management, rather than viewing them as competing or mutually exclusive strategies. Further studies with larger patient cohorts, standardized outcome measures, and extended follow-up periods remain essential to better characterize the role of regenerative medicine within multimodal epilepsy management and to delineate the precise contribution of individual therapeutic interventions to patient outcomes.
